# Postmenopausal Women with Breast, Endometrial, and Ovarian Cancers Have an Increased Risk for Cardiovascular Conditions prior to Active Endocrine Therapy

**DOI:** 10.1155/2022/5104351

**Published:** 2022-08-22

**Authors:** Xu Han, Fei Liu, Tesfaldet H. Hidru, Xiaolei Yang, Chengfang Wang, Yunlong Xia

**Affiliations:** ^1^Health Management Center, First Affiliated Hospital of Dalian Medical University, Dalian, Liaoning, China; ^2^Department of Cardiology, Institute of Cardiovascular Diseases, First Affiliated Hospital of Dalian Medical University, Dalian, Liaoning, China

## Abstract

**Background:**

Patients with active cancer have an increased risk of cardiovascular diseases (CVDs) among cancer patients receiving endocrine therapy. However, little research has explored the distribution of CVD comorbidities and cardiovascular risk factors (CVRFs) among postmenopause women with breast, endometrial, or ovarian cancer prior to active treatment with endocrine therapy. We aimed to explore the distribution of CVD comorbidities and associated CVRF in patients suffering from breast, endometrial, or ovarian cancer prior to the use of endocrine therapy and to assess whether there was compliance with existing hospital recommendations, particularly on the use of lipid-lowering agents to prevent the development of CVD comorbidities in postmenopause women.

**Methods:**

A total of 10,731 postmenopause women with primary breast, endometrial, or ovarian cancer were enrolled between 30th May 2008 and 31st July 2021 from an electronic health record database at the first affiliated hospital of Dalian Medical University. Dyslipidemia was defined according to 2016 Chinese guidelines for adults. Multivariate logistic regression analysis was used to identify the independent predictors of CVD comorbidities in breast, endometrial, and ovarian cancers separately.

**Results:**

Overall, 18.9% of the included women had at least one CVD record before endocrine therapy. The highest prevalence of CVD was identified for hypertension (16.5%), followed by coronary heart disease (4.5%), stroke (2.1%), heart failure (1.2%), and atrial fibrillation (1.1%). The most common CVRF among total cancer patients was dyslipidemia, with a remarkable prevalence of 62.8%, followed by diabetes mellitus (8.6%). Notably, only 11.1% of cancer patients were receiving lipid-lowering agents.

**Conclusion:**

Cancer patients with potential eligibility for endocrine therapy use had an increased risk for CVD comorbidities. Dyslipidemia was the common CVRF. Compliance with recommendations for preventing and managing these comorbidities requires serious attention.

## 1. Introduction

The data from the Global Burden of Disease Study 2017 indicates that cardiovascular disease (CVD) and cancer were the two leading causes of death globally [1]. The potential link between these two different disease entities has been well established. Recently, a scientific statement from the American Heart Association highlighted that cancer patients experience a significantly increased risk of CVD [2]. This may be the result of cancer treatment-related cardiotoxicity, which exerts adverse effects on heart function and/or structure. Therefore, given that cancer treatment-related cardiotoxicity is associated with adverse outcomes, there is a need to measure the risk of CVD before cancer patients are exposed to endocrine therapy.

The common agents of endocrine therapy including aromatase inhibitors (AIs) and Tamoxifen have significantly improved outcomes for postmenopausal female patients who suffered from breast cancer (BC) [3], endometrial cancer (EC) [4], and ovarian cancer (OC) [5]. However, it has recently been suggested that aromatase inhibitor users have an increased risk of subsequent CVD. Besides, postmenopausal women experience significant alterations in lipid profile due to the reduction in estrogen production. Therefore, an elaborate clinical assessment of CVD and CVRF before initiating endocrine therapy is of great importance in this subset of patients.

To the extent of our knowledge, data on the prevalence of cardiovascular-related comorbidities and CVRF among cancer patients eligible for endocrine therapy is limited. Therefore, this study sought to (1) explore the distribution of CVD comorbidities and associated CVRF in patients suffering from breast, endometrial, or ovarian cancer prior to the use of endocrine therapy and (2) assess whether there is compliance with existing hospital recommendations, particularly the use of lipid-lowering agents to prevent the development of these comorbidities in postmenopause women.

## 2. Materials

### 2.1. Study Design and Participants

This retrospective cross-sectional study was carried out at the first affiliated hospital of Dalian Medical University (FAHDM) among newly diagnosed cancer patients. We retrospectively evaluated the YiDuloud Electronic Medical Surveillance Network Database (YEMSND) at FAHDM from 30th May 2008 to 31st July 2021. Briefly, the YEMSND database was established to build a standardized clinical archive by updating clinical records continuously [6].

**Figure 1:**
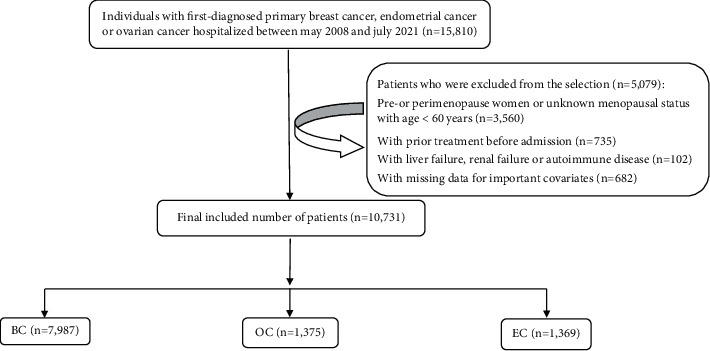
Flow chart of the study population. BC: breast cancer; EC: endometrial cancer; OC: ovarian cancer.

Inclusion criteria include postmenopausal women; histologically confirmed newly diagnosed primary breast, endometrial, or ovarian cancer; potential eligibility for endocrine therapy, and no previous use of anticancer therapy. A total of 15,810 histologically confirmed cancer patients were initially retrieved for this study. Of these, a total of 5079 patients were excluded. The excluded patients include women who were in premenopausal, perimenopause periods, or unknown menopausal status with age < 60 years (*n* = 3560); women with liver failure, renal failure, or autoimmune disease (*n* = 102); subjects with prior treatment before admission (*n* = 735); and patients with missing data for important covariates (*n* = 682). Finally, 10,731 cancer patients were included in the present study. The flow chart is given in Figure 1. The study was approved by the Ethics Committee of FAHDMU, and the committee waived the requirement of informed consent because our study utilized the recorded data from the hospital medical records.

### 2.2. Tumor Site Selection

The cancer types selected in this study were based on the potential eligibility for endocrine therapy in the future. Breast cancer, certain histologic subtypes of endometrial cancer, and ovarian cancer are hormone-dependent tumors in women, and the targeted therapy against hormone receptors has been identified as a powerful tool in the treatment of these cancers, leading to the inhibition of cell proliferation and cell-cycle arrest [7]. Recently, endocrine therapies have been recommended for breast cancer [8], endometrioid adenocarcinoma [9], and low-grade serous or grade 1 endometrioid ovarian cancer [10]. Therefore, historically confirmed breast cancer (BC), endometrial cancer (EC), and ovarian cancer (OC) were included in the current study.

### 2.3. Data Collection and Definition

Information on demographic characteristics (such as age, sex, height, weight, smoking, and drinking status), medication, and major CVRF and CVDs was summarized from YEMSND by professional medical staff. The retrieved CVRFs include the components of a lipid panel, plasma markers including fasting blood glucose (FPG), serum uric acid (SUA) and creatinine, and other risk factors such as systolic blood pressure (SBP), diastolic blood pressure (DBP), and diabetes mellitus (DM), whereas the major CVD diseases include hypertension (HTN), coronary heart disease (CHD), heart failure (HF), atrial fibrillation (AF), and stroke. Dyslipidemia was defined according to 2016 Chinese guidelines for the management of dyslipidemia in adults [11]. Briefly, patients were considered in a dyslipidemia state if they had total cholesterol (TC) > 6.2 mmol/L (240 mg/dL), low-density lipoprotein cholesterol (LDL‐C) ≥ 4.1 mmol/L (160 mg/dL), triglycerides (TG) ≥ 2.3 mmol/L (200 mg/dL), and high-density lipoprotein cholesterol (HDL‐C) ≤ 1.0 mmol/L (40 mg/dL) and/or if they were receiving a lipid-lowering agent. The non-high-density lipoprotein cholesterol (non-HDL-C) was calculated as TC minus HDL-C. The cut-off point to define normal, borderline high, and high levels of TC was <5.2 mmol/L, 5.2 ≤ TC < 6.2 mmol/L, and ≥6.2 mmol/L, respectively. DM was defined as FPG ≥ 7.0 mmol/L or a self-reported history of DM and/or treatment with antidiabetic medication. HTN was defined as SBP ≥ 140 mmHg or DBP ≥ 90 mmHg or a self-reported history of HTN and/or treatment with antihypertensive medication. CHD was defined based on the presence of either angina or coronary artery stenosis of 50% evidenced by medical records. HF was defined based on the clinical symptoms, echocardiography, chest X-ray, and electrocardiography [12]. AF was diagnosed if one of the following criteria was met: (1) AF pattern in 10 s 12-lead electrocardiogram (ECG), (2) AF episodes in 24-hour Holter, or (3) a self-reported history of AF. As per the hospital protocol, two independent experienced cardiologists who were blind to the clinical data validated all the ECG readings. Stroke was defined as an acute episode of focal dysfunction of the brain, retina, or spinal cord lasting longer than 24 h or of any duration if imaging (CT or MRI) or autopsy shows focal infarction or hemorrhage relevant to the symptoms [13]. The current smoker was defined as current smoking status or a lifetime consumption of more than 100 cigarettes [14]. One drink was defined as an average of 15 g of ethanol, and drinking for women was defined as at least 1 drink/day in the past year [15]. Body mass index (BMI) was calculated as weight (kg) divided by the square of height (m^2^). The 10-year Framingham risk score (FRS) was used to evaluate the risk for cardiovascular disease, which was classified as low (<10%), moderate (10-20%), or high (>20%) risk [14].

### 2.4. Statistical Analysis

Normally distributed continuous variables were presented as the mean ± standard deviation (SD) and compared using ANOVA analysis in three or more groups. The independent-sample *t*-test was used to compare the differences in demographic and clinical characteristics between the dyslipidemia and nondyslipidemia groups. Categorical variables were presented as percentages and computed for differences using the *χ*^2^ test. Multivariate logistic regression analysis was used to calculate the odds ratio (OR) and 95% confidence interval (CI) and identify the predictors of CVD comorbidities in BC, EC, and OC. The model was adjusted for age, SBP, TC, TG, HDL-C, SUA ≥ 360 *μ*mol/L, and DM. All statistical tests were 2-sided, and *P* value < 0.05 was considered statistically significant. The statistical analysis was performed using SPSS version 25.0 software (SPSS, Chicago, Illinois, USA).

## 3. Results

### 3.1. Baseline Characteristics of the Participants

Among 10,731 women included, BC, OC, and EC account for 7987 (74.43%), 1375 (12.81%), and 1369 (12.75%). The mean age of the study participants was 61.49 years at cancer diagnosis. We found that 18.9% of postmenopausal women with cancer suffered from at least one CVD. Overall, the highest prevalence of CVD was identified for hypertension (16.5%), followed by coronary heart disease (4.5%), stroke (2.1%), heart failure (1.2%), and atrial fibrillation (1.1%). The most common CVRF among total cancer patients was dyslipidemia (62.8%), followed by DM (8.6%). Only 11.1% of the cancer patients received a prescription for lipid-lowering agents.

### 3.2. Cardiovascular-Related Comorbidity Prevalence among Diverse Cancers

The comparison of basic characteristics of cancer patients is presented in Table 1. Among postmenopausal women with cancer, the two common comorbidities in EC patients were HTN and DM with prevalence rates of 24.8% and 13.4%, respectively. The BC and OC patients had a higher prevalence of stroke than EC patients. The prevalence rates of stroke in BC, OC, and EC were 2.3%, 2.2%, and 1%, respectively. There was no significant difference in the prevalence of CHD, HF, and AF among the three different types of cancer.

### 3.3. Common Cardiovascular Risk Factors in Cancer Patients

Dyslipidemia was the most crucial CVRF among the three types of cancer. About 62.8% of the included women experienced different degrees of lipid metabolic disorders. The prevalence of dyslipidemia in BC, EC, and OC patients was 61.3%, 66.8%, and 67.5%, respectively. EC patients had the highest levels of SBP, DBP, BMI, FPG, and SUA. The proportion of DM patients in BC, EC, and OC was 7.8%, 13.4%, and 8.8%, respectively. When 10-year FHS risk was calculated, 9.8% of the included patients recorded high FHS risk. The rate of high 10-years FHS risk was 9.1%, 14.5%, and 8.9% in BC, EC, and OC, respectively.

### 3.4. The Distribution of Dyslipidemia and Lipid Indices in BC, OC, and EC

Table 2 shows the distribution of various cholesterol indicators among different cancers. The overall prevalence of dyslipidemia among total cancer patients was 62.8%, and the prevalence of elevated TC, elevated TG, elevated LDL-C, and decreased HDL-C was 16.0%, 14.6%, 7.3%, and 44.1%, respectively. The patients who suffered from BC had the highest prevalence of increased TC (17.4%), increased LDL-C (7.6%), increased TC+increased TG (4.8%), increased TC+increased LDL-C (7.2%), and increased TG+increased LDL-C (2.2%) compared to EC and OC patients. We found that decreased HDL-C was the most common dyslipidemia among OC patients, with a prevalence of 56.6%. Also, we observed a substantial proportion of increased TG (17.2%), increased TC+decreased HDL-C (2.8%), increased TG+decreased HDL-C (9.6%), and increased LDL-C and decreased HDL-C (2.0%) in EC patients (17.2%). BC patients had the highest mean levels of TC, LDL-C, and non-HDL-C. EC patients had the highest mean levels of TG. Importantly, OC patients had the lowest mean levels of HDL-C.

### 3.5. The Distribution of Blood Lipid Components and Use of Lipid-Lowering Agents

To understand the primary prevention of atherosclerotic cardiovascular disease (ASCVD), we further evaluated the serum lipid status based on standard classification criteria. As shown in Table 3, only 53.9%, 74.4%, 62.6%, and 70.1% of the enrolled cancer patients reached the recommended TC, LDL, non-HDL, and TG levels, indicating that a significant proportion of patients failed to achieve the target value. Similar observations occurred in BC, EC, and OC patients. The proportion of substandard lipid control in BC patients was the highest among total cancer patients.

**Figure 2:**
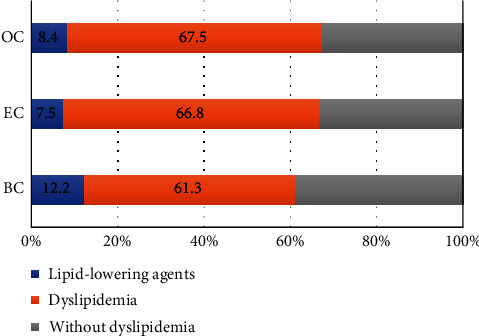
Distribution of dyslipidemia and proportion of receiving lower-lipid agents among different cancer patients. BC: breast cancer; EC: endometrial cancer; OC: ovarian cancer.

Figure 2 presents the distribution of dyslipidemia and the use of lipid-lowering agents among the studied cancers. The proportion of dyslipidemia was the highest in EC patients. However, the use of lipid-lowering agents was the lowest (7.5%) among EC patients. A similar observation was observed for OC (8.4%) and EC (12.2%), indicating a low prescription rate of lipid-lowering agents.

### 3.6. Coprevalence of Dyslipidemia with Other CVDs and CVRF

Table 4 presents the comparison of CVD and CVRF between the dyslipidemia and nondyslipidemia groups. Compared with nondyslipidemia, individuals with dyslipidemia were more likely to be older and had a higher proportion of DM. The mean values of FPG, SBP, DBP, and creatinine were significantly higher in patients with dyslipidemia. The mean level of SUA was higher in the dyslipidemia group except for OC patients.

In addition, cancer patients with disturbed lipid metabolism, indicated by abnormal levels of lipid indicators, were found to often suffer from a wide range of cardiovascular comorbidities, including HTN, CHD, HF, and stroke. The prevalence of HTN was higher in the dyslipidemia group compared to the nondyslipidemia group except for OC patients. There was no difference in the distribution of AF in the dyslipidemia group and the nondyslipidemia group among BC, EC, and OC patients.

### 3.7. Risk Factors for CVDs in BC, OC, and EC

Table 5 presents the results of multivariate logistic regression analysis for conventional cardiovascular risk factors associated with CVDs in BC, OC, and EC. We observed a positive relationship between DM and the presence of CVDs in all selected cancers. Participants with DM had a higher likelihood of CVDs compared with non-DM patients. The OR and 95% CI for BC, EC, and OC patients were 9.12 (7.54-11.05), 9.04 (6.31-12.94), and 5.43 (3.59-8.23), respectively.

The multivariate logistic regression model showed that advanced age and high levels of SUA (≥360 *μ*mol/L) were independent risk factors for CVDs among all cancers. Also, we observed that BC patients with high levels of TG had a higher likelihood of CVDs (OR: 1.10; 95% CI: 1.05-1.16, *P* < 0.001). Likewise, higher levels of HDL-C were associated with a lower prevalence of CVDs in EC patients (OR: 0.60; 95% CI: 0.36-0.99, *P* = 0.048).

## 4. Discussion

The present study demonstrated that the prevalence of CVD and CVRF was high in BC, EC, and OC. Dyslipidemia dominates other CVRFs. Cancer patients with disturbed lipid metabolism were more likely to have cardiovascular comorbidities compared to patients with normal lipid indices. The rate of lipid-lowering prescription was significantly low compared to the rate of dyslipidemia in cancer patients, which requires special attention by the oncologists to adhere to the existing hospital recommendations to control lipid levels.

In the present study, 62.8% of women in the postmenopausal stage had dyslipidemia. In line with our study, He et al. reported that the prevalence of preoperative hyperlipidemia was 60.27% in postmenopausal patients [16]. This indicates that dyslipidemia is increasingly common among women with cancer who are at the postmenopausal stage. According to our results, the prevalence of elevated levels of TC, LDL-C, TG, and HDL-C hypolipidemia was 16.0%, 7.3%, 14.6%, and 44.1%, respectively. These results are higher than the previously reported prevalence of lipid indicators among the general population [17, 18]. The age- and sex-related hormonal differences may explain the observed discrepancy in lipid metabolism between the general population and postmenopausal women.

According to the present study, HDL-C hypolipidemia was the most common dyslipidemia among OC patients, with a prevalence of 56.6%. An earlier study has also revealed that metabolic disturbances of serum lipids, blood glucose, and inflammatory response are more prominent in OC patients, with the results showing lower levels of serum TC and HDL-C in OC patients than those of the control group [19]. On the contrary, Delimaris et al. observed no statistically significant difference in serum levels of TC, LDL-C, and HDL-C between patients diagnosed with OC and healthy individuals [20]. The discrepancy may be partly explained by the difference in body mass index among OC patients. The study by Delimaris et al. included only 15 OC patients, and all were nonoverweight patients.

Our study also observed a significant proportion of dyslipidemia (61.3%), elevated TC (17.4%), and decreased HDL-C (40.7%) in BC patients. Similarly, a study conducted among Taiwanese women demonstrated that BC patients had significantly lower HDL-C, apolipoprotein A-I (apoA-I), and apoA-I/apolipoprotein B (apoB) ratios and higher very-low-density lipoprotein cholesterol than controls [21]. However, a retrospective cohort study on the status of lipid and lipoprotein among BC patients before adjuvant chemotherapy found low levels of TC, TG, LDL-C, and HDL-C and decreased prevalence of dyslipidemia in the BC group [22]. Further longitudinal study is required to explain the reason that causes discrepancies in blood lipid levels among BC patients.

Endocrine therapy used for BC, EC, and OC can cause significant alterations in serum lipid profiles after treatment [23]. For instance, the estrogen agnostic effects of Tamoxifen lower TC and LDL-C [24]. However, a previous study reported an increase in serum triglyceride levels among patients using Tamoxifen [25], which could further lead to hypertriglyceridemia [26]. In the present study, the overall prevalence of increased TG before treatment was 14.6%, and patients who suffered from EC had the highest prevalence of increased TG (17.2%) compared to BC and OC patients. Hence, the assessment, control, and management of dyslipidemia and increased TG prior to active cancer treatment with endocrine therapy are of great importance.

Akin to Mazzutti et al. [27], our results reported a high frequency of systemic arterial hypertension. Also, in this study, a significant proportion of postmenopause women with cancer were classified as medium and high 10-year cardiovascular risk according to FHS. Furthermore, 18.9% of the postmenopausal female patients suffered from at least one CVD before they received their endocrine therapy. In the past, Abdel-Qadir et al. observed that women diagnosed with an early stage of BC were more likely to have a history of heart failure, ischemic heart disease, cerebrovascular disease, peripheral vascular disease, atrial fibrillation, and hypertension compared to cancer-free controls [28]. As such, given the high risk for cardiovascular events (as indicated by FHS scores in our study) and the increased risk of subsequent cardiovascular disease, including heart failure, myocardial infarction, and stroke due to endocrine agents such as Ais [29], proper assessment of the modifiable CVRFs and CVDs before initiating endocrine therapy should not be underestimated.

Many studies highlighted the intimate relationship between dyslipidemia and various cancers. Lipid metabolism abnormality plays a crucial role in the prognosis of cancers. As earlier mentioned, our study demonstrated the disturbance of the lipid indicators, though the disturbance in the lipid metabolism varies based on the cancer type. In contrast to the high burden of dyslipidemia, we noticed that a relatively low percentage of postmenopausal women with cancer were receiving lipid-lowering agents before initiating active cancer treatment. This indicates that more work is required to achieve optimal lipid control among cancer patients. Therefore, proper utilization of statins should be encouraged to lower the risk of CVD, because positive outcomes have been noted among statin users in BC [30], EC [31], and OC [32] patients. A recent Chinese expert consensus recommends practical strategies for clinicians in the management of dyslipidemia in malignant tumors [33]. Thus, patients with the potential eligibility for endocrine therapy (especially hormone receptor-positive) should be advised to regularly check their lipid profiles.

According to Li et al., the association between DM and the presence of CVDs was strong in breast, lung, colorectal, and gastric cancers [34]. Similarly, the present study demonstrated that BC, OC, and EC patients diagnosed with DM had a significantly increased risk for CVDs. Not only is DM and disturbed lipid metabolism increasingly associated with CVD risk in cancer patients but they are also common risk factors for CVD in the general population. However, whether the risk projected by DM and other lipid indicators is similar to the general population remained unknown. Substantiated clinical data is required to weigh the effect of abnormal lipid indicators on the mechanism of CVD in cancer patients.

In the present study, an elevated level of SUA (≥360 *μ*mol/L) was found to be an independent risk factor for CVD. The link that connects SUA, CVD, and cancer is complex and could be mediated by several factors such as diet, chronic inflammatory burden, and oxidative stress. Among the many factors, inflammation is a vital and common factor that links CVD and cancer. Interestingly, increased levels of SUA can generate inflammatory stress by producing reactive oxygen species (ROS)/reactive nitrogen species (RNS) and activating cyclo-oxygenase-2 (COX-2) [35]. In addition, a recent study suggested that hyperuricemia promotes atherosclerosis by disturbing the balance of the asymmetric dimethylarginine (ADMA)/dimethylarginine dimethylaminotransferase-2 (ADMA/DDAH-2) axis [36]. Earlier evidence also concluded that oxidative stress and its direct consequences including lipid peroxidation promote the pathophysiological changes during the pathogenesis of atherosclerosis, cancer, and inflammation [37].

This is the first study that assessed the distribution of CVD-related comorbidities among postmenopausal women before the use of endocrine treatment. However, the present work has some limitations. First, the database was not supportive to retrieve detailed information on the stage of the tumor. Second, the prevalence of CVD-associated comorbidities may be underestimated in our study, although rigorous retrieval procedure has been made by trained medical staff. Third, our single-center study was conducted in a coastal city in north China; thus, the participants cannot be viewed as a representative sample of the general Chinese population.

## 5. Conclusions

In conclusion, our findings demonstrate that cancer patients with potential eligibility for endocrine therapy carry a significant burden of cardiovascular diseases and cardiovascular risk factors. The prevalence of dyslipidemia was the highest among various CVRFs. Considering the importance of controlling serum lipid levels to achieve positive outcomes, oncologists and cardiologists should cooperate closely with each other and provide appropriate strategies for the prevention, detection, and management of dyslipidemia and other cardiovascular risks prior to endocrine therapy in postmenopausal women with cancer.

## Figures and Tables

**Table 1:** The baseline characteristics of the participants (*n* = 10,731).

Variables	Total (*n* = 10,731)	Breast cancer (*n* = 7987)	Endometrial cancer (*n* = 1375)	Ovarian cancer (*n* = 1369)	*P* value
Age (years)	61.49 ± 12.60	61.01 ± 12.63	63.62 ± 11.38	61.12 ± 13.31	<0.001
Smoking, *N* (%)	95 (0.9%)	68 (0.9)	12 (0.9)	15 (1.1)	0.671
Drinking, *N* (%)	44 (0.4)	36 (0.5)	5 (0.4)	3 (0.2)	0.445
SBP (mmHg)	128.66 ± 17.44	128.59 ± 17.44	132.00 ± 17.00	125.70 ± 17.29	<0.001
DBP (mmHg)	79.47 ± 19.91	79.36 ± 9.79	80.99 ± 10.17	78.54 ± 10.19	<0.001
BMI (kg/m^2^)	23.88 ± 3.75	23.79 ± 3.59	24.93 ± 4.22	23.38 ± 3.92	<0.001
HTN, *N* (%)	1767 (16.5)	1149 (14.4)	341 (24.8)	277 (20.2)	<0.001
CHD, *N* (%)	482 (4.5)	375 (4.7)	50 (3.6)	57 (4.2)	0.177
HF, *N* (%)	129 (1.2)	94 (1.2)	12 (0.9)	23 (1.7)	0.140
AF, *N* (%)	117 (1.1)	87 (1.1)	17 (1.2)	13 (0.9)	0.770
DM, *N* (%)	927 (8.6)	622 (7.8)	184 (13.4)	121 (8.8)	<0.001
Stroke, *N* (%)	226 (2.1)	182 (2.3)	14 (1.0)	30 (2.2)	<0.001
Dyslipidemia	6739 (62.8)	4896 (61.3)	919 (66.8)	924 (67.5)	<0.001
TC (mmol/L)	5.15 ± 1.13	5.21 ± 1.14	5.08 ± 1.09	4.89 ± 1.06	<0.001
TG (mmol/L)	1.57 ± 1.16	1.59 ± 1.21	1.64 ± 1.11	1.39 ± 0.94	<0.001
LDL-C (mmol/L)	2.94 ± 0.80	2.96 ± 0.80	2.95 ± 0.80	2.84 ± 0.75	<0.001
HDL-C (mmol/L)	1.28 ± 0.35	1.30 ± 0.35	1.22 ± 0.33	1.18 ± 0.32	<0.001
Non-HDL-C (mmol/L)	3.87 ± 0.97	3.90 ± 0.99	3.86 ± 0.94	3.72 ± 0.92	<0.001
FPG (mmol/L)	5.57 ± 1.75	5.70 ± 1.71	6.09 ± 2.03	5.63 ± 1.62	<0.001
SUA (*μ*mol/L)	285.13 ± 76.12	281.65 ± 73.37	300.83 ± 81.44	289.68 ± 83.83	<0.001
Creatinine (*μ*mol/L)	54.51 ± 11.90	54.15 ± 11.01	54.87 ± 12.30	56.28 ± 15.73	<0.001
Lipid-lowering agents, *N* (%)	1196 (11.1)	978 (12.2)	103 (7.5)	115 (8.4)	<0.001
Diuretics	1422 (13.3)	885 (11.1)	125 (9.1)	412 (30.1)	<0.001
ACEI/ARB	1074 (10.0)	777 (9.7)	168 (12.2)	129 (9.4)	<0.013
*β*-Blockers	1160 (10.8)	872 (10.9)	122 (8.9)	166 (12.1)	<0.019
CCB	2134 (19.9)	1501 (18.8)	349 (25.4)	284 (20.7)	<0.001
FHS risk, low	6663 (62.1)	5096 (63.8)	696 (50.6)	871 (63.6)	<0.001
Moderate	3020 (28.1)	2164 (27.1)	480 (34.9)	376 (27.5)	
High	1048 (9.8)	727 (9.1)	199 (14.5)	122 (8.9)	

AF: atrial fibrillation; CHD: coronary heart disease; DM: diabetes mellitus; DBP: diastolic blood pressure; FHS: Framingham Heart Study; FPG: fasting plasma glucose; HDL-C: high-density lipoprotein cholesterol; HF: heart failure; HTN: hypertension; LDL-C: low-density lipoprotein cholesterol; non-HLD-C: non-high-density lipoprotein cholesterol; SBP: systolic blood pressure; SUA: serum uric acid; TC: total cholesterol; TG: triglycerides.

**Table 2:** Prevalence of dyslipidemia among cancer patients.

*N* (%)	Total	Breast cancer	Endometrial cancer	Ovarian cancer	*P* value
Dyslipidemia	6739 (62.8)	4896 (61.3)	919 (66.8)	924 (67.5)	<0.001
Elevated TC	1719 (16.0)	1388 (17.4)	187 (13.6)	144 (10.5)	<0.001
Elevated TG	1526 (14.6)	1199 (15.0)	236 (17.2)	127 (9.3)	<0.001
Elevated LDL-C	780 (7.3)	608 (7.6)	102 (7.4)	70 (5.1)	<0.001
Decreased HDL-C	4730 (44.1)	3249 (40.7)	706 (51.3)	775 (56.6)	<0.001
Elevated TC+elevated TG	479 (4.5)	382 (4.8)	61 (4.4)	36 (2.6)	0.002
Elevated TC+elevated LDL-C	739 (6.9)	579 (7.2)	95 (6.9)	65 (4.7)	0.003
Elevated TC+decreased HDL-C	224 (2.1)	150 (1.9)	38 (2.8)	36 (2.6)	0.034
Elevated TG+elevated LDL-C	217 (2.0)	172 (2.2)	26 (1.9)	19 (1.4)	0.166
Elevated TG+decreased HDL-C	678 (6.3)	484 (6.1)	132 (9.6)	62 (4.5)	<0.001
Elevated LDL-C+decreased HDL-C	126 (1.2)	81 (1.0)	27 (2.0)	18 (1.3)	0.009

HDL-C: high-density lipoprotein cholesterol; LDL-C: low-density lipoprotein cholesterol; TC: total cholesterol; TG: triglycerides.

**Table 3:** The serum lipid control among cancer patients.

*N* (%)	Total	Breast cancer	Endometrial cancer	Ovarian cancer	*P* value
*TC (mmol/L)*					<0.001
Appropriate level, TC < 5.2	5782 (53.9)	4134 (51.8)	775 (56.4)	873 (63.8)	
Borderline high, 5.2 ≤ TC < 6.2	3171 (29.5)	2419 (30.3)	408 (29.7)	344 (25.1)	
High, TC ≥ 6.2	1778 (16.6)	1434 (18.0)	192 (14.0)	152 (11.1)	
*LDL (mmol/L)*					<0.001
Ideal level, LDL < 2.6	3702 (34.5)	2696 (33.8)	465 (33.8)	541 (39.5)	
Appropriate level, 2.6 ≤ LDL < 3.4	4277 (39.9)	3153 (39.5)	559 (40/7)	565 (41.3)	
Borderline high, 3.4 ≤ LDL < 4.1	1928 (18.0)	1496 (18.7)	243 (17.7)	189 (13.8)	
High LDL, ≥4.1 L	824 (7.7)	642 (8.0)	108 (7.9)	74 (5.4)	
*HDL (mmol/L)*					<0.001
Low HDL, <1.0	4730 (44.1)	3249 (40.7)	706 (51.3)	775 (56.6)	
*Non-HDL (mmol/L)*					<0.001
Ideal level, non-HDL < 3.4	3340 (32.1)	2475 (31.0)	432 (31.4)	533 (38.9)	
Appropriate level, non-HDL < 4.1	3269 (30.5)	2418 (30.3)	426 (31.0)	425 (31.0)	
Borderline high, 4.1 ≤ non‐HDL < 4.9	2568 (23.9)	1958 (24.5)	344 (25.0)	266 (19.4)	
High, non-HDL ≥ 4.9	1454 (13.5)	1136 (14.2)	173 (12.6)	145 (10.6)	
*TG (mmol/L)*					<0.001
Appropriate level, TG < 1.7	7518 (70.1)	5520 (69.1)	921 (67.0)	1077 (78.7)	
Borderline high, 1.7 ≤ TG < 2.3	1721 (16.0)	1319 (16.5)	228 (16.6)	174 (12.7)	
High, TG ≥ 2.3	1492 (13.9)	1148 (14.4)	226 (16.4)	118 (8.6)	

HDL-C: high-density lipoprotein cholesterol; LDL-C: low-density lipoprotein cholesterol; non-HLD-C: non-high-density lipoprotein cholesterol; TC: total cholesterol; TG: triglycerides.

**Table 4:** Comparison of cardiovascular diseases and cardiovascular risk factors between dyslipidemia and without dyslipidemia groups.

Variables	Total		Breast cancer		Endometrial cancer		Ovarian cancer	
	Dyslipidemia	Without dyslipidemia	Dyslipidemia	Without dyslipidemia	Dyslipidemia	Without dyslipidemia	Dyslipidemia	Without dyslipidemia
Age (years)	61.78 ± 12.53	60.99 ± 12.70^&^	61.57 ± 12.60	60.13 ± 12.63^∗^	63.86 ± 11.82	63.25 ± 11.15	63.71 ± 13.19	61.36 ± 13.31^§^
Smoking, *N* (%)	57 (0.8)	38 (1.0)	41 (0.8)	27 (0.9)	7 (0.8)	5 (1.1)	9 (1.0)	6 (1.3)
Drinking, *N* (%)	28 (0.4)	16 (0.4)	21 (0.4)	15 (0.5)	4 (0.4)	1 (0.2)	3 (0.3)	0 (0.0)
SBP (mmHg)	130.59 ± 17.56	127.29 ± 16.60^&^	130.61 ± 17.65	127.56 ± 17.02^∗^	133.72 ± 17.69	130.56 ± 16.64^£^	127.45 ± 17.44	124.35 ± 16.26^§^
DBP (mmHg)	80.43 ± 9.96	78.53 ± 9.83^&^	80.39 ± 9.81	78.32 ± 9.76^∗^	82.04 ± 10.30	79.90 ± 9.93^£^	79.55 ± 10.09	78.05 ± 10.12^§^
HTN, *N* (%)	1195 (17.7)	572 (14.3)^&^	760 (15.5)	389 (12.6)^∗^	245 (26.7)	96 (21.1)^£^	190 (20.6)	87 (19.6)
CHD, *N* (%)	322 (4.8)	160 (4.0)^&^	246 (5.0)	129 (4.2)^∗^	35 (3.8)	15 (3.3)^£^	41 (4.4)	16 (3.6)^§^
HF, *N* (%)	95 (1.4)	34 (0.9)^&^	68 (1.4)	26 (0.8)^∗^	8 (0.9)	4 (0.9)	19 (2.1)	4 (0.9)^§^
AF, *N* (%)	75 (1.1)	42 (1.1)	55 (1.1)	32 (1.0)	8 (0.9)	4 (0.9)	15 (1.6)	8 (1.8)
DM, *N* (%)	631 (9.4)	296 (7.4)^&^	412 (8.4)	210 (6.8)^∗^	131 (14.3)	53 (11.6)^£^	88 (9.5)	33 (7.4)^§^
Stroke, *N* (%)	160 (2.4)	66 (1.7)^&^	122 (2.5)	60 (1.9)^∗^	12 (1.3)	2 (0.4)^£^	26 (2.8)	4 (0.9)^§^
Glu (mmol/L)	5.84 ± 1.84	5.59 ± 1.57^&^	5.80 ± 1.81	5.56 ± 1.52^∗^	6.22 ± 2.06	5.84 ± 1.95^£^	5.68 ± 1.70	5.53 ± 1.44^§^
SUA (*μ*mol/L)	290.38 ± 78.07	275.51 ± 71.69^&^	287.71 ± 75.49	272.05 ± 68.79^∗^	307.43 ± 82.90	287.53 ± 76.80^£^	290.87 ± 84.33	287.22 ± 82.81
Creatinine (*μ*mol/L)	54.73 ± 12.57	54.14 ± 11.58^&^	54.39 ± 11.58	53.75 ± 10.02^∗^	54.99 ± 12.90	54.65 ± 10.98	56.28 ± 16.54	56.30 ± 13.91

&, ∗, £, and § mean *P* < 0.05 among total and different cancers when compared to the group with dyslipidemia and without dyslipidemia. AF: atrial fibrillation; CHD: coronary heart disease; DM: diabetes mellitus; DBP: diastolic blood pressure; FPG: fasting plasma glucose; HF: heart failure; HTN: hypertension; SBP: systolic blood pressure; SUA: serum uric acid.

**Table 5:** Cardiovascular risk factors in BC, EC, and OC.

Cancer sites	CVRFs	*B*	SE	Wald	OR	95% CI	*P*
BC	Age	0.066	0.003	477.176	1.068	1.061-1.074	<0.001
	SUA ≥ 360 *μ*mol/L	0.481	0.087	30.709	1.618	1.365-1.918	<0.001
	TG	0.095	0.025	13.960	1.100	1.046-1.156	<0.001
	DM	2.211	0.098	512.460	9.124	7.535-11.049	<0.001

EC	Age	0.035	0.006	32.226	1.036	1.023-1.040	<0.001
	SUA ≥ 360 *μ*mol/L	0.722	0.160	20.407	2.059	1.505-2.816	<0.001
	HDL-C	-0.515	0.261	3.908	0.597	0.358-0.996	0.048
	TC	0.146	0.078	3.510	1.158	0.993-1.349	0.061
	DM	2.201	0.183	144.206	9.037	6.309-12.944	<0.001

OC	Age	0.056	0.006	81.044	1.058	1.045-1.071	<0.001
	SUA ≥ 360 *μ*mol/L	0.646	0.172	14.153	1.907	1.362-2.670	<0.001
	TG	0.130	0.069	3.537	1.139	0.995-1.304	0.060
	DM	1.692	0.212	63.829	5.431	3.586-8.225	<0.001

BC: breast cancer; CI: confidence interval; CVRFs: cardiovascular risk factors; DM: diabetes mellitus; EC: endometrial cancer; HDL-C: high-density lipoprotein cholesterol; OC: ovarian cancer; OR: odds ratio; SE: standard error; SUA: serum uric acid; TC: total cholesterol; TG: triglycerides. Adjusted for age, SBP, SUA ≥ 360 *μ*mol/L, TC, TG, HDL-C, and DM.

## Data Availability

The data of this study were retrieved and extracted from the YiDuloud Electronic Medical Surveillance Network Database at the first affiliated hospital of Dalian Medical University (FAHDM), but restrictions apply to the availability of these data, which were used under license for the current study, and so are not publicly available.

## References

[1] G. B. D. C. o. D. Collaborators (2018). Global, regional, and national age-sex-specific mortality for 282 causes of death in 195 countries and territories, 1980-2017: a systematic analysis for the Global Burden of Disease Study 2017. *Lancet*.

[2] Mehta L. S., Watson K. E., Barac A. (2018). Cardiovascular disease and breast cancer: where these entities intersect: a scientific statement from the American Heart Association. *Circulation*.

[3] Burstein H. J., Prestrud A. A., Seidenfeld J. (2010). American Society of Clinical Oncology clinical practice guideline: update on adjuvant endocrine therapy for women with hormone receptor-positive breast cancer. *Journal of Clinical Oncology*.

[4] Slomovitz B. M., Jiang Y., Yates M. S. (2015). Phase II study of everolimus and letrozole in patients with recurrent endometrial carcinoma. *Journal of Clinical Oncology*.

[5] Ray-Coquard I., Morice P., Lorusso D. (2018). Non-epithelial ovarian cancer: ESMO Clinical Practice Guidelines for diagnosis, treatment and follow-up^†^. *Annals of Oncology*.

[6] Liu F., Hidru T. H., Gao R. (2020). Cancer patients with potential eligibility for vascular endothelial growth factor antagonists use have an increased risk for cardiovascular diseases comorbidities. *Journal of Hypertension*.

[7] Dellapasqua S., Colleoni M., Gelber R. D., Goldhirsch A. (2005). Adjuvant endocrine therapy for premenopausal women with early breast cancer. *Journal of Clinical Oncology*.

[8] Gradishar W. J., Anderson B. O., Abraham J. (2020). Breast cancer, version 3.2020, NCCN clinical practice guidelines in oncology. *Journal of the National Comprehensive Cancer Network*.

[9] Koh W. J., Abu-Rustum N. R., Bean S. (2018). Uterine neoplasms, version 1.2018, NCCN clinical practice guidelines in oncology. *Journal of the National Comprehensive Cancer Network*.

[10] Armstrong D. K., Alvarez R. D., Bakkum-Gamez J. N. (2021). Ovarian cancer, version 2.2020, NCCN clinical practice guidelines in oncology. *Journal of the National Comprehensive Cancer Network*.

[11] Jun-Ren Z. H., Run-Lin G. A., Shui-Ping Z. H., Guo-Ping L. U., Dong Z. H., Jian-Jun L. I. (2018). 2016 Chinese guidelines for the management of dyslipidemia in adults. *Journal of Geriatric Cardiology*.

[12] Ponikowski P., Voors A. A., Anker S. D. (2016). 2016 ESC guidelines for the diagnosis and treatment of acute and chronic heart failure: the task force for the diagnosis and treatment of acute and chronic heart failure of the European Society of Cardiology (ESC) developed with the special contribution of the Heart Failure Association (HFA) of the ESC. *European Heart Journal*.

[13] Hankey G. J. (2017). Stroke. *Lancet*.

[14] Ford E. S., Giles W. H., Mokdad A. H. (2004). The distribution of 10-year risk for coronary heart disease among U.S. adults: findings from the National Health and Nutrition Examination Survey III. *Journal of the American College of Cardiology*.

[15] Li Z., Bai Y., Guo X., Zheng L., Sun Y., Roselle A. M. (2016). Alcohol consumption and cardiovascular diseases in rural China. *International Journal of Cardiology*.

[16] He T., Wang C., Tan Q. (2020). Adjuvant chemotherapy-associated lipid changes in breast cancer patients: a real-word retrospective analysis. *Medicine (Baltimore)*.

[17] Xi Y., Niu L., Cao N. (2020). Prevalence of dyslipidemia and associated risk factors among adults aged >/=35 years in northern China: a cross-sectional study. *BMC Public Health*.

[18] Lin H. Q., Wu J. Y., Chen M. L. (2019). Prevalence of dyslipidemia and prediction of 10-year CVD risk among older adults living in southeast coastal regions in China: a cross-sectional study. *Clinical Interventions in Aging*.

[19] Li G., Zhang K., Gong F., Jin H. (2019). A study on changes and clinical significance of blood glucose, blood lipid and inflammation in patients with ovarian cancer. *Journal of BUON*.

[20] Delimaris I., Faviou E., Antonakos G., Stathopoulou E., Zachari A., Dionyssiou-Asteriou A. (2007). Oxidized LDL, serum oxidizability and serum lipid levels in patients with breast or ovarian cancer. *Clinical Biochemistry*.

[21] Chang S. J., Hou M. F., Tsai S. M. (2007). The association between lipid profiles and breast cancer among Taiwanese women. *Clinical Chemistry and Laboratory Medicine*.

[22] Li X., Liu Z. L., Wu Y. T. (2018). Status of lipid and lipoprotein in female breast cancer patients at initial diagnosis and during chemotherapy. *Lipids in health and disease*.

[23] Esteva F. J., Hortobagyi G. N. (2006). Comparative assessment of lipid effects of endocrine therapy for breast cancer: implications for cardiovascular disease prevention in postmenopausal women. *Breast*.

[24] Dewar J. A., Horobin J. M., Preece P. E., Tavendale R., Tunstall-Pedoe H., Wood R. A. (1992). Long term effects of tamoxifen on blood lipid values in breast cancer. *BMJ*.

[25] Hozumi Y., Kawano M., Saito T., Miyata M. (1998). Effect of tamoxifen on serum lipid metabolism. *The Journal of Clinical Endocrinology and Metabolism*.

[26] Liu C. L., Yang T. L. (2003). Sequential changes in serum triglyceride levels during adjuvant tamoxifen therapy in breast cancer patients and the effect of dose reduction. *Breast Cancer Research and Treatment*.

[27] Mazzutti F. S., Custódio I. D., Lima M. T. (2021). Breast cancer survivors undergoing endocrine therapy have a worrying risk factor profile for cardiovascular diseases. *Nutrients*.

[28] Abdel-Qadir H., Thavendiranathan P., Austin P. C. (2019). The risk of heart failure and other cardiovascular hospitalizations after early stage breast cancer: a matched cohort study. *Journal of the National Cancer Institute*.

[29] Khosrow-Khavar F., Filion K. B., Bouganim N., Suissa S., Azoulay L. (2020). Aromatase inhibitors and the risk of cardiovascular outcomes in women with breast cancer: a population-based cohort study. *Circulation*.

[30] Moksud N., Loo L. W. M., Yang J. (2021). Cholesterol lowering drug use and breast cancer survival: the multiethnic cohort study. *Breast Cancer Research and Treatment*.

[31] Sperling C. D., Verdoodt F., Kjaer Hansen M., Dehlendorff C., Friis S., Kjaer S. K. (2018). Statin use and mortality among endometrial cancer patients: a Danish nationwide cohort study. *International Journal of Cancer*.

[32] Yarmolinsky J., Bull C. J., Vincent E. E. (2020). Association between genetically proxied inhibition of HMG-CoA reductase and epithelial ovarian cancer. *JAMA*.

[33] A. Integrative Cardio-Oncology Society of China Anti-Cancer (2021). Chinese expert consensus on lipid management in patients with malignancy. *Zhonghua Zhong Liu Za Zhi*.

[34] Li Q., Liu F., Tang Y. (2021). The distribution of cardiovascular-related comorbidities in different adult-onset cancers and related risk factors: analysis of 10 year retrospective data. *Frontiers in cardiovascular medicine*.

[35] Che X. H., Chen C. L., Ye X. L. (2016). Dual inhibition of COX-2/5-LOX blocks colon cancer proliferation, migration and invasion in vitro. *Oncology Reports*.

[36] Lee T. S., Lu T. M., Chen C. H., Guo B. C., Hsu C. P. (2021). Hyperuricemia induces endothelial dysfunction and accelerates atherosclerosis by disturbing the asymmetric dimethylarginine/dimethylarginine dimethylaminotransferase 2 pathway. *Redox Biology*.

[37] Barrera G. (2012). Oxidative stress and lipid peroxidation products in cancer progression and therapy. *International Scholarly Research Notices*.

